# Higher breast cancer prevalence associated with higher socioeconomic status in the South Korean population; Has it resulted from overdiagnosis?

**DOI:** 10.1371/journal.pone.0200484

**Published:** 2018-07-12

**Authors:** Seong-Woo Choi, So-Yeon Ryu, Mi-ah Han, Jong Park

**Affiliations:** Department of Preventive Medicine, Chosun University Medical School, 309, Pilmun-daero, Dong-gu, Gwangju, Republic of Korea; Universidad Miguel Hernandez de Elche, SPAIN

## Abstract

Recently, breast cancer prevalence has increased in South Korea. In this study, we investigated the correlation between breast cancer prevalence and socioeconomic status. This study enrolled 27,331 people who participated in the Korean National Health and Nutrition Examination Survey (KNHANES) IV–VI (2007–2015). In addition, we obtained data from the Korean Statistical Information Service (KSIS) on the breast cancer age-standardized incidence rate (AIR), the age-standardized mortality rate (AMR), the number of women screened, and the number of newly diagnosed patients. The KHANES data showed that breast cancer prevalence was significantly associated with educational level (odds ratio [OR], 2.02; 95% confidence interval [CI], 1.08–3.77 for 10–12 vs. ≤ 6 years of education, and OR, 2.36; 95% CI, 1.10–5.06 for ≥ 13 vs. ≤ 6 years of education). However, there was no significant association of breast cancer prevalence with monthly household income. In a separate analysis of the AIR, AMR, and number of women screened for breast cancer, the AIR increased with the number of women screened, whereas the AMR did not. Furthermore, the number of newly diagnosed patients in all age groups increased over time. The present results demonstrate that the recently increased breast cancer prevalence documented in South Korea may be attributable to earlier detection rather than to a real increase in prevalence, and that breast cancer may be overdiagnosed.

## Introduction

Cancer is one of the leading causes of death worldwide; 8.8 million patients died from cancer in 2015 [1]. Several risk factors are known, including smoking, alcohol consumption, obesity, an unhealthy diet, infectious agents, and environmental carcinogens [2]. In addition, socioeconomic status (SES) affects cancer rates [3]. A low SES was associated with higher cancer incidence and mortality [4, 5]. However, some studies have suggested that a higher SES is a risk factor for diagnosis of some cancers because high SES individuals may undergo more frequent health check-ups [6, 7].

In South Korea, the thyroid cancer incidence rate has increased rapidly over the past two decades [8]. It has been suggested that this reflects both overdiagnosis and overtreatment [8, 9]. However, no concern has yet been raised about overdiagnosis of breast cancer in South Korea; newly diagnosed patients with breast cancer have increased by greater than threefold in the past 15 years [10]. Many studies in western countries have found relationships between a higher risk of breast cancer and a higher SES [11–14]. However, breast cancer risks vary by ethnicity, social culture, and the healthcare system [15]. Thus, the relationship between SES and breast cancer must be assessed on a country-by-country basis. To the best of our knowledge, only one prior study has been conducted in Korea, which reported that a lower SES was a risk factor for breast cancer [6], thus contradicting previous studies performed in other countries. However, the generalizability of the results was limited because the participants were not representative of the entire Korean population.

Thus, we examined the correlation between breast cancer prevalence and SES using two data sources: 1) the Korean National Health and Nutrition Examination Survey (KNHANES) IV–VI (2007–2015) for data on SES, health related behaviors, and healthcare utilization; and 2) the Korean Statistical Information Service (KSIS) for data on the breast cancer age-standardized incidence rate (AIR), the age-standardized mortality rate (AMR), the number of women screened, and the number of newly diagnosed patients.

## Materials and methods

### Subjects

We conducted this study using KNHANES IV–VI (2007–2015) data. The Korea Centers for Disease Control and Prevention (KCDCP) conducted the KNHANES, which uses a nationally representative, cross-sectional design [16]. KNHANES surveys Ⅰ, Ⅱ, and Ⅲ were performed in 1998, 2001, and 2005, respectively. Since KNHANES IV, the survey has been conducted annually (IV, 2007–2009; Ⅴ, 2010–2012; and VI, 2013–2015). The KNHANES consisted of health interviews, questionnaires, a physical examination, and a nutrition survey. Health interviews and examinations are performed in mobile centers equipped to allow the undertaking of health interviews and examinations. A total of 73,353 subjects were enrolled in KNHANES IV–VI (2007–2015) (2007: 4,594; 2008: 9,744; 2009: 10,533; 2010: 8,958; 2011: 8,518; 2012: 8,058; 2013: 8,018; 2014: 7,550; and 2015: 7,380). Our current survey excluded 45,235 respondents (males: 33,337; females aged ≤ 19 years: 8,555; absence of cancer data: 2,526; absence of sociodemographic or behavioral data: 1,624). Therefore, we analyzed a total of 27,311 subjects in this study (see S1 Dataset). All subjects provided informed consent for use of their data. Our study was conducted in accordance with the guidelines of the Declaration of Helsinki. The KCDCP ethics committee approved the study protocol (2007-02CON-04-P, 2008-04EXP-01-C, 2009-01CON-03-2C, 2010-02CON-21-C, 2011-02CON-06-C, 2012-01EXP-01-2C, 2013-07CON-03-4C, 2014-12EXP-03-5C, 2015-01-02-6C).

### Study measurements

As described in detail previously [17], all subjects were interviewed by trained examiners using a questionnaire exploring monthly household income, years of education, marital status, residential area, smoking habits, alcohol intake, physical activity, history of cancer examination, and diagnosis of breast cancer. Trained researchers measured the weight (to the nearest 0.1 kg), height, and waist circumference (both to the nearest 0.1 cm) of the subjects while they were wearing light clothing and socks. The waist circumference (WC) was measured at expiration using a flexible tape, positioned parallel to the floor at the midpoint between the iliac crest and the lowest rib.

The smoking status was assessed by self-report; subjects who had smoked more than 100 cigarettes in their lifetime were classified as past or current smokers based on their current smoking habits. Alcohol intake in the past month was assessed by exploring drinking behavior during the month prior to interview. Physical activity was coded “yes” when a subject walked for > 30 min more than five times per week. Cancer examination in the prior 2 years was coded “yes” when a subject had undergone cancer screening within the prior 2 years for any type of cancer, including breast, lung, colon, skin, and ovarian cancer, etc. Previous pregnancy was coded “yes” for subjects had ever been pregnant, including normal pregnancy, ectopic pregnancy, still birth, spontaneous abortion, and induced abortion. The prevalence of breast cancer was measured by asking: “Before study enrollment, were you ever diagnosed by a doctor with breast cancer?” We classified those who answered “yes” as having breast cancer.

Subjects were divided into monthly household income quartiles: low (≤ 1,200,000 won), medium-low (1,210,000–2,500,000 won), medium-high (2,510,000–4,300,000 won), and high (≥ 4,310,000 won). Subjects were also classified by educational level: ≤ 6 years, 7–9 years, 10–12 years, and ≥ 13 years.

We obtained the breast cancer AIR and AMR, the numbers of women screened, and the numbers of newly diagnosed patients, from the KSIS [10].

### Statistical analysis

We used SAS software version 9.3 (SAS Inc., Cary, NC, USA) for the statistical analyses. The survey responses were weighted by reference to the multistage, complex, probability sampling design. Data were expressed as estimated percentages (with standard errors [SEs]) and the distributions of monthly household income and educational level were assessed by the χ^2^ test. The odds ratios (ORs) with 95% confidence intervals (CIs) for the association of breast cancer prevalence with potential risk factors were compared using a multiple logistic regression model. Age and survey year were included as confounders in Model 1. Waist circumference, marital status, residential area, current smoking, alcohol use in the past month, physical activity, cancer examination in the prior 2 years, previous pregnancy, current or past use of oral contraceptives, post-menopausal, and age at menarche were adjusted for in Model 2, in addition to the variables included in Model 1. Educational level or monthly household income and the variables included in Model 2 were adjusted for in Model 3. A *p*-value < 0.05 was deemed to reflect statistical significance.

## Results

### Baseline characteristics

A total of 236 had breast cancer. In terms of marital status, 15.6% were single and 84.4% married. With respect to residential area, 81.4% and 18.6% lived in urban and rural areas, respectively. Of all the subjects, 7.2% currently smoked and 66.1% consumed alcohol in the past month; 83.2% had ever been pregnant and 15.5% were currently using, or had used oral contraceptives in the past (Table 1).

**Table 1:** Baseline characteristics of subjects.

Variables	*N*	e%(SE)
Number	27,311	
Breast cancer patients	236	0.8(0.1)
Age (year)		
19–44	10,893	48.4(0.5)
45–64	9,942	35.3(0.4)
65–74	6,476	16.4(0.3)
Waist circumference (cm)		
<80	15,240	59.8(0.4)
80–90	5,283	27.9(0.4)
>90	3,688	12.2(0.3)
Survey year		
2007	1,450	5.1(0.5)
2008	3,455	10.7(0.7)
2009	3,783	11.1(0.7)
2010	3,408	12.1(0.8)
2011	3,381	12.5(0.8)
2012	3,199	12.2(0.8)
2013	3,024	12.3(0.8)
2014	2,818	11.7(0.8)
2015	2,793	12.2(0.8)
Monthly household income (10,000 won)		
Lowest (≤120)	6,926	20.4(0.4)
Medium-lowest (121–250)	6,764	25.6(0.4)
Medium-highest (251–430)	6,805	26.9(0.4)
Highest (≥431)	6,816	27.0(0.5)
Education (year)		
≤6	8,751	24.7(0.4)
7–9	2,841	10.0(0.2)
10–12	8,568	35.5(0.4)
≥13	7,151	29.8(0.4)
Marital status		
Single	2,974	15.6(0.4)
Married	24,337	84.4(0.4)
Residential area		
Urban	21,335	81.4(0.9)
Rural	5,976	18.6(0.9)
Current smoking	1,734	7.2(0.2)
Alcohol intake in past month	16,823	66.1(0.4)
Physically active^a^	10,469	38.7(0.4)
Cancer examination in the prior 2 years^b^	15,968	55.8(0.4)
Previous pregnancy^c^	23,944	83.2(0.4)
Current or past use of oral contraceptive	4,508	15.5(0.3)
Post-menopausal	7,029	20.3(0.5)
Age at menarche (year)		
≤12	3,878	17.8(0.3)
13–14	9,569	37.7(0.4)
15–16	7,876	27.4(0.3)
≥17	5,599	17.1(0.3)

All values are given as number and estimated percentage (standard error).

^a^Physically active was indicated as ‘yes’ when the subject walked for more than 30 min at a time and more than five times per week.

^b^Cancer examination in the prior 2 years was indicated as ‘yes’ when the subject had undergone screening within the prior 2 years for any kind of cancer such as lung cancer, stomach cancer, and breast cancer etc.

^c^Previous pregnancy was indicated as ‘yes’ when the a subject had ever been pregnant including normal pregnancy, ectopic pregnancy, still birth, spontaneous abortion, and induced abortion.

### Characteristics of subjects by quartile of monthly household income

Higher monthly household income tended to be associated with younger age, lower WC, a higher educational level, being single, residing in urban areas, a lower likelihood of being a current smoker, higher alcohol intake in the past month, completion of a higher number of cancer examinations in the prior 2 years, less experience of pregnancy, less use of oral contraceptives, pre-menopausal status, and a younger age at menarche (Table 2).

**Table 2:** Characteristics of subjects by quartile of monthly household income.

	Monthly household income (10,000 won)								
Variables	Lowest (≤120)		Medium-lowest (121–250)		Medium-highest (251–430)		Highest (≥431)		p-value
	N	e%(SE)	N	e%(SE)	N	e%(SE)	N	e%(SE)	
Breast cancer prevalence (%)	68	0.9(0.1)	57	0.7(0.1)	51	0.7(0.1)	60	0.8(0.1)	0.504
Age (year)									<0.001
19–44	756	18.1(0.7)	2,887	51.3(0.8)	3,715	60.1(0.7)	3,535	56.6(0.7)	
45–64	2,121	32.6(0.7)	2,679	36.9(0.7)	2,395	32.7(0.6)	2,747	38.2(0.7)	
65–74	4,049	49.3(0.8)	1,198	11.8(0.4)	695	7.2(0.3)	534	5.1(0.3)	
Waist circumference (cm)									<0.001
<80	2,777	43.2(0.8)	3,631	58.0(0.8)	4,232	64.1(0.7)	4,600	70.0(0.7)	
80–90	2,601	35.9(0.7)	2,132	28.9(0.7)	1,871	26.0(0.6)	1,679	22.9(0.6)	
>90	1,511	20.9(0.7)	977	13.1(0.5)	682	9.9(0.4)	518	7.1(0.4)	
Survey year									<0.001
2007	500	6.9(0.9)	401	6.1(0.7)	374	5.5(0.7)	175	2.6(0.4)	
2008	1,132	14.0(1.0)	1,022	13.4(1.0)	800	10.0(0.8)	501	6.4(0.7)	
2009	1,136	13.6(1.0)	1,086	13.3(0.9)	890	10.5(0.8)	671	7.9(0.8)	
2010	702	10.8(1.0)	842	12.5(1.0)	950	13.1(1.0)	914	11.7(1.0)	
2011	752	11.0(1.0)	736	11.6(0.9)	850	12.6(1.0)	1,043	14.4(1.2)	
2012	754	11.3(1.0)	761	12.3(1.1)	801	12.4(1.0)	883	12.4(1.1)	
2013	688	11.4(1.0)	692	10.9(0.9)	790	12.8(1.0)	854	13.8(1.2)	
2014	666	10.8(0.9)	620	10.0(0.8)	716	12.0(1.0)	816	13.7(1.3)	
2015	596	10.1(0.9)	604	10.0(0.8)	634	11.0(0.9)	959	17.1(1.4)	
Education (year)									<0.001
≤6	4,942	63.1(0.9)	1,966	23.0(0.6)	1,098	13.3(0.5)	745	8.5(0.4)	
7–9	701	10.8(0.5)	947	13.0(0.5)	679	9.4(0.4)	514	7.2(0.4)	
10–12	927	18.6(0.7)	2,482	40.7(0.8)	2,724	41.6(0.7)	2,435	37.3(0.8)	
≥13	356	7.5(0.4)	1,369	23.2(0.7)	2,304	35.7(0.8)	3,122	47.1(0.8)	
Marital status									<0.001
Single	390	9.8(0.6)	731	15.0(0.6)	831	16.5(0.6)	1,022	19.5(0.6)	
Married	6,536	90.2(0.6)	6,033	85.0(0.6)	5,974	83.5(0.6)	5,794	80.5(0.6)	
Residential area									<0.001
Urban	4,518	71.2(1.4)	5,256	80.6(1.1)	5,668	84.0(1.1)	5,893	87.3(1.0)	
Rural	2,408	28.8(1.4)	1,508	19.4(1.1)	1,137	16.0(1.1)	923	12.7(1.0)	
Current smoking	581	9.9(0.5)	539	9.3(0.5)	364	6.1(0.4)	250	4.3(0.4)	<0.001
Alcohol intake in past month	3,074	48.7(0.8)	4,205	65.8(0.7)	4,655	71.0(0.7)	4,889	74.6(0.6)	<0.001
Physically active^a^	2,622	37.5(0.7)	2,684	40.3(0.7)	2,610	38.6(0.7)	2,553	38.1(0.7)	0.047
Cancer examination in the prior 2 years^b^	3,766	51.8(0.8)	3,829	53.6(0.7)	3,991	55.8(0.7)	4,400	60.9(0.7)	<0.001
Previous pregnancy^c^	6,446	89.7(0.6)	5,946	84.0(0.7)	5,867	81.9(0.6)	5,685	78.8(0.6)	<0.001
Current or past use of oral contraceptive	1,502	20.8(0.6)	1,225	16.9(0.6)	951	13.6(0.5)	830	11.9(0.5)	<0.001
Post-menopausal	3,270	40.1(1.0)	1,635	18.8(0.6)	1,106	13.3(0.5)	1,018	13.6(0.6)	<0.001
Age at menarche (year)									<0.001
<13	400	9.2(0.5)	938	17.2(0.6)	1,181	19.9(0.6)	1,359	22.7(0.7)	
13–14	1,444	24.1(0.7)	2,370	37.4(0.7)	2,784	41.6(0.7)	2,971	44.2(0.7)	
15–16	2,271	32.4(0.7)	2,036	28.6(0.7)	1,860	26.2(0.6)	1,709	23.8(0.6)	
≥17	2,607	34.4(0.8)	1,337	16.7(0.5)	919	12.2(0.5)	736	9.4(0.4)	

All values are given as number and estimated percentage (standard error).

^a^Physically active was indicated as ‘yes’ when the subject walked for more than 30 min at a time and more than five times per week.

^b^Cancer examination in the prior 2 years was indicated as ‘yes’ when the subject had undergone screening within the prior 2 years for any kind of cancer such as lung cancer, stomach cancer, and breast cancer etc.

^c^Previous pregnancy was indicated as ‘yes’ when the a subject had ever been pregnant including normal pregnancy, ectopic pregnancy, still birth, spontaneous abortion, and induced abortion.

### Characteristics of subjects by educational level

Higher educational level tended to be associated with younger age, lower WC, higher monthly household income, being single, residing in urban areas, higher alcohol intake in the past month, less previous pregnancy, less use of oral contraceptives, pre-menopausal status, and a younger age at menarche (Table 3).

**Table 3:** Characteristics of subjects by education level.

Variables	Education (year)								
	≤6		7–9		10–12		≥13		p-value
	N	e%(SE)	N	e%(SE)	N	e%(SE)	N	e%(SE)	
Breast cancer prevalence (%)	87	1.0(0.1)	32	1.0(0.2)	67	0.7(0.1)	50	0.6(0.1)	0.051
Age (year)									<0.001
19–44	106	2.0(0.2)	324	14.5(0.9)	4,850	62.0(0.7)	5,613	81.8(0.6)	
45–64	3,278	42.5(0.7)	2,011	72.6(1.0)	3,256	34.9(0.6)	1,397	17.1(0.6)	
65–74	5,367	55.5(0.7)	506	12.9(0.7)	462	3.1(0.2)	141	1.1(0.1)	
Waist circumference (cm)									<0.001
<80	3,027	35.2(0.7)	1,302	47.2(1.2)	5,473	65.9(0.6)	5,438	77.2(0.6)	
80–90	3,618	41.0(0.6)	1,078	37.4(1.1)	2,281	25.1(0.6)	1,306	17.3(0.5)	
>90	2,064	23.7(0.6)	452	15.4(0.8)	783	9.0(0.4)	389	5.5(0.3)	
Survey year									<0.001
2007	529	5.5(0.7)	156	5.6(0.7)	457	5.5(0.6)	308	4.2(0.6)	
2008	1,241	11.6(0.8)	356	11.1(0.9)	1,100	11.2(0.8)	758	9.2(0.8)	
2009	1,316	12.4(0.9)	399	11.3(0.9)	1,225	11.7(0.8)	843	9.4(0.8)	
2010	1,091	13.4(1.2)	341	12.2(1.2)	1,047	11.3(0.9)	929	12.0(0.9)	
2011	1,093	12.9(1.1)	356	12.9(1.2)	1,043	12.3(0.9)	889	12.2(1.0)	
2012	1,009	11.9(1.0)	335	12.5(1.1)	994	12.7(1.0)	861	11.7(1.0)	
2013	882	11.9(1.0)	299	11.3(1.0)	974	12.1(0.9)	869	13.3(1.1)	
2014	810	10.1(0.8)	286	11.1(1.0)	879	11.6(0.9)	843	13.4(1.1)	
2015	780	10.2(0.8)	313	12.0(1.1)	849	11.6(0.9)	851	14.7(1.2)	
Monthly household income (10,000 won)									<0.001
Lowest (≤120)	4,942	52.2(0.7)	701	21.9(1.0)	927	10.7(0.5)	356	5.1(0.3)	
Medium-lowest (121–250)	1,966	24.0(0.6)	947	33.3(1.1)	2,482	29.4(0.7)	1,369	20.0(0.6)	
Medium-highest (251–430)	1,098	14.6(0.5)	679	25.4(1.0)	2,724	31.5(0.6)	2,304	32.2(0.7)	
Highest (≥431)	745	9.3(0.4)	514	19.4(1.0)	2,435	28.4(0.7)	3,122	42.7(0.9)	
Marital status									<0.001
Single	41	0.4(0.1)	38	1.9(0.3)	1,148	17.7(0.6)	1,747	30.2(0.8)	
Married	8,710	99.6(0.1)	2,803	98.1(0.3)	7,420	82.3(0.6)	5,404	69.8(0.8)	
Residential area									<0.001
Urban	5,460	67.2(1.5)	2,211	79.4(1.4)	7,258	84.9(1.0)	6,406	89.6(0.8)	
Rural	3,291	32.8(1.5)	630	20.6(1.4)	1,310	15.1(1.0)	745	10.4(0.8)	
Current smoking	536	6.6(0.3)	195	8.6(0.7)	708	9.4(0.4)	295	4.6(0.3)	<0.001
Alcohol intake in past month	3,718	45.1(0.7)	1,721	64.4(1.1)	6,156	73.6(0.6)	5,228	75.0(0.6)	<0.001
Physically active^a^	3,183	35.2(0.7)	1,131	39.9(1.1)	3,498	41.4(0.7)	2,657	37.8(0.7)	<0.001
Cancer examination in the prior 2 years^b^	5,065	57.6(0.7)	1,899	65.0(1.1)	5,013	54.6(0.7)	4,009	52.7(0.7)	<0.001
Previous pregnancy^c^	8,591	98.7(0.1)	2,784	97.9(0.3)	7,347	81.7(0.6)	5,222	67.2(0.8)	<0.001
Current or past use of oral contraceptive	2,040	23.2(0.6)	646	22.4(1.0)	1,161	13.3(0.4)	661	9.4(0.4)	<0.001
Post-menopausal	4,368	45.5(1.1)	1,014	32.5(1.2)	1,167	11.7(0.5)	480	5.4(0.4)	<0.001
Age at menarche (year)									<0.001
<13	254	3.8(0.3)	156	6.8(0.6)	1,432	19.1(0.5)	2,036	31.2(0.7)	
13–14	1,302	16.3(0.5)	800	28.4(1.0)	3,953	46.2(0.6)	3,514	47.9(0.7)	
15–16	3,104	36.5(0.7)	1,063	37.6(1.1)	2,379	26.5(0.6)	1,330	17.7(0.6)	
≥17	3,791	43.4(0.7)	797	27.2(1.0)	769	8.2(0.3)	242	3.1(0.2)	

All values are given as number and estimated percentage (standard error).

^a^Physically active was indicated as ‘yes’ when the subject walked for more than 30 min at a time and more than five times per week.

^b^Cancer examination in the prior 2 years was indicated as ‘yes’ when the subject had undergone screening within the prior 2 years for any kind of cancer such as lung cancer, stomach cancer, and breast cancer etc.

^c^Previous pregnancy was indicated as ‘yes’ when the a subject had ever been pregnant including normal pregnancy, ectopic pregnancy, still birth, spontaneous abortion, and induced abortion.

### The ORs for breast cancer prevalence by quartile of monthly household income

When adjusted for age and survey year (Model 1), the breast cancer prevalence was significantly associated with monthly household income (OR, 1.67; 95% CI, 1.02–2.72, highest vs. lowest quartile). However, after additionally adjusting for WC, marital status, residential area, current smoking, alcohol intake in the past month, physical activity, cancer examination in the past 2 years, previous pregnancy, current or past use of oral contraceptives, post-menopausal status, age at menarche (Model 2) and educational level (Model 3), the associations between breast cancer prevalence and monthly household income were attenuated and no longer statistically significant. (Table 4).

**Table 4:** The ORs for breast cancer prevalence by quartile of monthly household income.

	Model 1^a^	Model 2^b^	Model 3^c^
	OR(95%CI)	OR (95%CI)	OR(95%CI)
Monthly household income			
medium-lowest/lowest	1.44 (0.92–2.24)	1.33 (0.85–2.09)	1.23 (0.78–1.95)
medium-highest/lowest	1.43 (0.86–2.36)	1.32 (0.77–2.25)	1.16 (0.67–2.02)
highest/lowest	1.67 (1.02–2.72)	1.49 (0.90–2.45)	1.25 (0.74–2.11)

^a^Adjusted by age and survey year

^b^Adjusted by Model 1 variables plus waist circumference, marital status, residential area, current smoking, alcohol intake in the past month, physical activity, cancer examination in the past 2 years, previous pregnancy, current or past use of oral contraceptives, post-menopausal status and age at menarche

^c^Adjusted by Model 2 variables plus education level

### The ORs for breast cancer prevalence by educational level

When adjusted for age and the survey year (Model 1), breast cancer prevalence was significantly associated with educational level (OR, 1.92; 95% CI, 1.11–3.32 for 7–9 years vs. ≤ 6 years; OR, 2.32; 95% CI, 1.34–4.09 for 10–12 years vs. ≤ 6 years; OR, 2.87; 95% CI, 1.44–5.70 for ≥ 13 years vs. ≤ 6 years). When also adjusted for WC, marital status, residential area, current smoking status, alcohol intake in the past month, physical activity, cancer examination during the past 2 years, previous pregnancy, current or past use of oral contraceptives, post-menopausal status, and age at menarche (Model 2), the associations between breast cancer prevalence and educational level remained significant (OR, 1.96; 95% CI, 1.10–3.50 for 10–12 years vs. ≤ 6 years; OR, 2.36; 95% CI, 1.16–4.80 for ≥ 13 years vs. ≤ 6 years). When additionally adjusted for monthly household income (Model 3), the associations remained significant (OR, 1.86; 95% CI, 1.03–3.38 for 10–12 years vs. ≤ 6 years, and OR, 2.22; 95% CI, 1.06–4.65 for ≥ 13 years vs. ≤ 6 years) (Table 5).

**Table 5:** The ORs for breast cancer prevalence by education level.

	Model 1^a^	Model 2^b^	Model 3^c^
	OR(95%CI)	OR (95%CI)	OR(95%CI)
Education (year)			
7-9/≤6	1.92 (1.11–3.32)	1.64 (0.94–2.86)	1.58 (0.88–2.83)
10-12/≤6	2.32 (1.32–4.09)	1.96 (1.10–3.50)	1.86 (1.03–3.38)
≥13/≤6	2.87 (1.44–5.70)	2.36 (1.16–4.80)	2.22 (1.06–4.65)

^a^Adjusted by age and survey year

^b^Adjusted by Model 1 variables plus waist circumference, marital status, residential area, current smoking status, alcohol intake in the past month, physical activity, cancer examination during the past 2 years, previous pregnancy, current or past use of oral contraceptives, post-menopausal status, and age at menarche

^c^Adjusted by Model 2 variables plus monthly household income

### The AIR, AMR, and the numbers of women screened for breast cancer in South Korea

**Fig 1:**
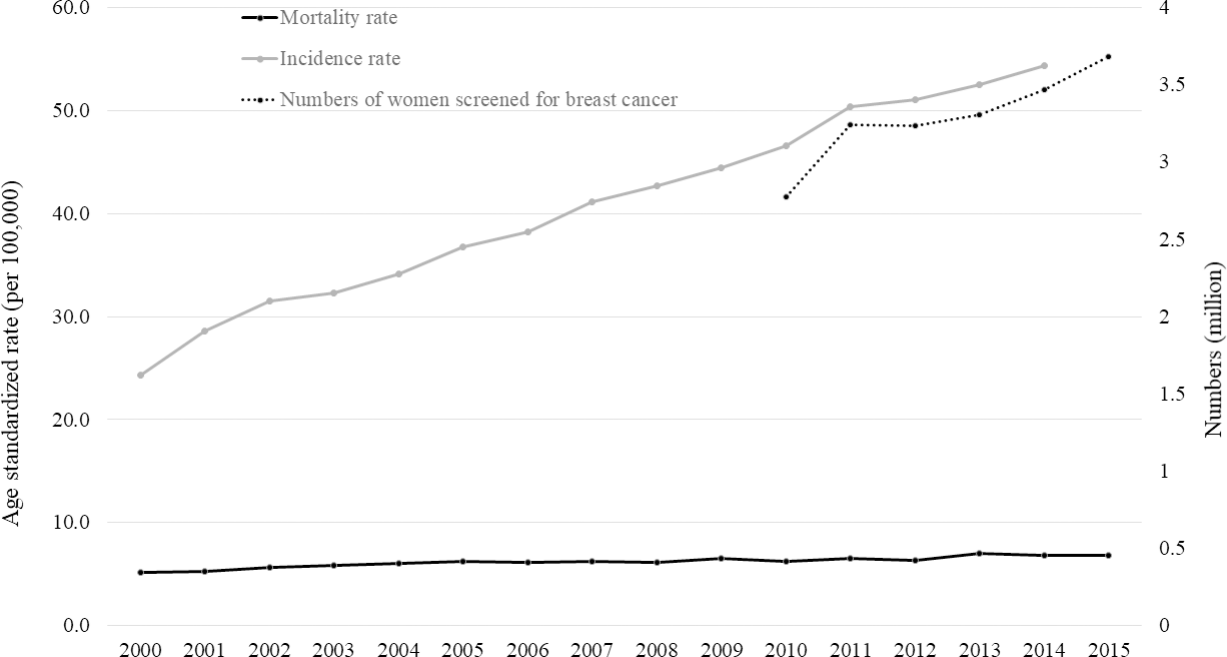
Age-standardized breast cancer incidence and mortality rate, and the number of women screened for breast cancer in South Korea from 2000–2015.

Fig 1 shows the AIR, AMR, and the numbers of women screened for breast cancer in South Korea (2000–2015). The AIR of breast cancer increased 2.2-fold (24.3 per 100,000 in 2000; 54.4 per 100,000 in 2014) over this time. However, the AMR of breast cancer remained largely unchanged (5.2 per 100,000 in 2000; 6.8 per 100,000 in 2014). The number of women screened increased from 2.88 million in 2010 to 3.68 million in 2015.

### Trends over time in newly diagnosed breast cancer by age group

**Fig 2:**
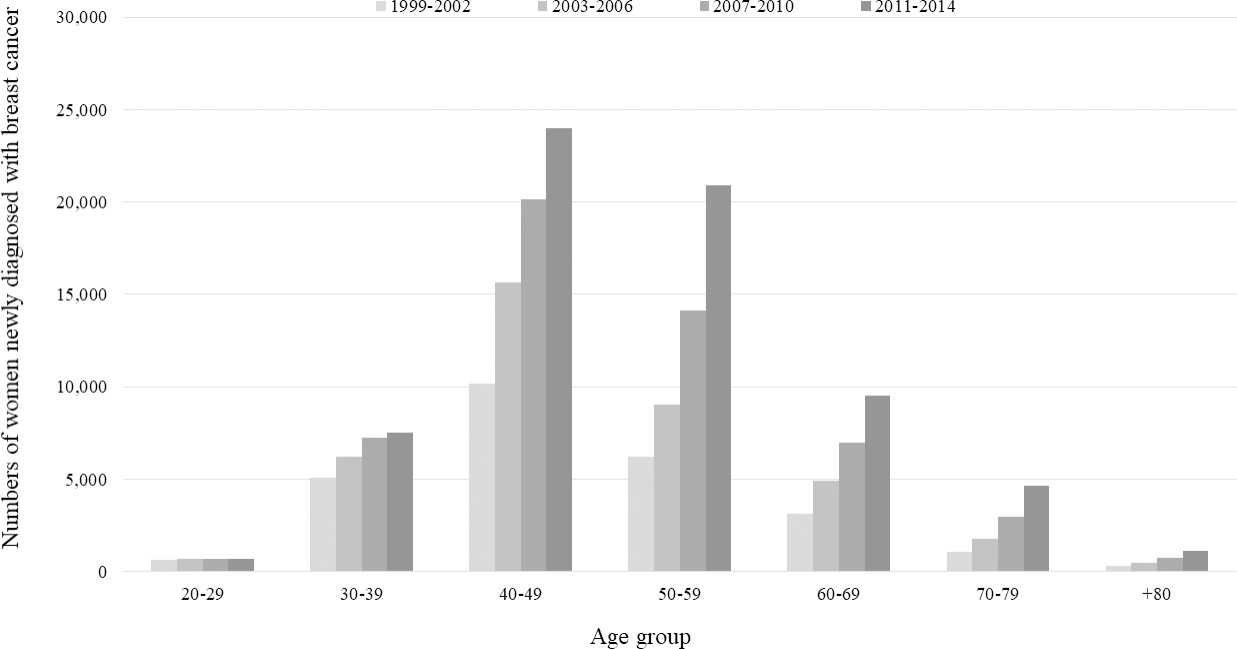
Trends over time in the number of women newly diagnosed with breast cancer by age group.

Fig 2 shows the trends over time in newly diagnosed breast cancer by age group. The number of newly diagnosed patients was highest among those aged 40–49 years, and decreased at higher ages. Also, the numbers of newly diagnosed patients in all age groups increased over time.

## Discussion

We examined the relationship between breast cancer prevalence and SES using KNHANES IV–VI (2007–2015) and KSIS data. Our results demonstrated that higher educational level was significantly associated with breast cancer prevalence after adjusting for other covariates.

In Western countries, it is well established that higher SES is associated with an elevated incidence of breast cancer [11–14]. However, breast cancer risks vary by ethnicity, reproductive practices, cancer screening, cultural factors, and the healthcare system [15, 18, 19]. Thus, the relationship between SES and breast cancer must be assessed on a country-by-country basis. To the best of our knowledge, only one prior study has been conducted in Korea [6]. This work used data from the cancer registry of the National Health Insurance Corporation (NHIC) to examine the association between SES and cancer incidence. Surprisingly, a lower income was a risk factor for breast cancer, which was not consistent with previous results. However, the generalizability of the results is limited because the participants were not representative of the entire Korean population; they included only medical aid beneficiaries and the self-employed. Moreover, SES was gaged by reference to family income only.

Our results demonstrated that breast cancer prevalence was not associated with monthly household income, whereas it was associated with educational level. This difference may be due to the Korean national cancer screening program; the government introduced the program for the entire Korean population in 1999 to cover five cancers: stomach, breast, colorectal, cervical, and liver. Furthermore, cancer screening is also provided free of charge for the poorest in society, and is of very low cost for the rest of the population [20]. In a previous study using a national cancer screening survey [21], breast cancer prevalence in Korea was not associated with household income, but did show an association with educational level. However, for thyroid cancer [17], which was excluded from the national cancer screening program, prevalence was significantly associated with both income and educational level.

In our present study, we found that a higher SES was associated with a greater prevalence of breast cancer. Several plausible explanations may be advanced. First, the well-known risks for breast cancer including nulliparity, late maternal age at first birth, and use of hormone replacement therapies and oral contraceptives, are all associated with a higher SES [22–26]. However, we found that even after adjustment for covariates including reproductive practices, a significant association between SES and breast cancer remained. Second, as individuals of higher SES have greater access to healthcare services [27], and are more often screened [21, 28, 29], a higher detection rate might contribute to the greater prevalence of breast cancer in the higher SES group. Also, the AIR of breast cancer seems to reflect the number of females screened (Fig 1), rendering it very likely that the rise in breast cancer in Korea is attributable to increased detection.

The relationship between a higher SES and a greater prevalence of breast cancer deserves discussion. Socioeconomic disparities in breast cancer status (incidence, screening, stage at diagnosis, treatment, survival, and mortality) are apparent [21, 30]. However, it must be asked whether breast cancer is overdiagnosed [31–33]. Specifically, no consensus has emerged on the age at which regular screening mammography should commence. The U.S. Preventive Services Task Force has recommended that females commence regular biennial screening mammography at the age of 50 rather than 40 years [34]. However, the American Cancer Society has strongly recommended that females aged 45–54 years should be screened annually [35]. The Korean national cancer screening program funds biennial screening mammography for all Korean females > 40 years [36]. However, this commencement age was chosen not on the basis of evidence from Korean females, but by reference to data gathered in other countries including the United States, Canada, and certain European countries [20]. Some of our data suggest that breast cancer is overdiagnosed during screening in Korea. First, the rapid increase in the AIR, but the stability of the AMR, over 15 years (Fig 1) constitutes pathognomonic evidence of overdiagnosis [37]. Second, we assessed the trends over time in the numbers of newly diagnosed patients by age group, and found something of interest. If mammographic screening of those aged 40–49 years was effective, such screening should advance the time of diagnosis [38], and the breast cancer incidence in females aged ≥ 50 years should decline over time. However, the incidence has actually increased (Fig 2). Third, the previous study that used Korean national cancer screening data [39] found that the positive predictive value of the breast cancer screening program was only 0.6%, indicating that less than 1 female in 100 of those initially suspected to have cancer actually had cancer. Breast cancer screening was thus both ineffective and overdiagnostic.

Several years ago, both public health experts and major Korean newspapers spoke out about thyroid cancer overdiagnosis [8, 40]. As a result, and for the first time, the incidence of thyroid cancer and number of thyroid operations decreased markedly in 2014 [41], as did the incidence of prostate cancer [10]. However, the breast cancer incidence did not change, perhaps because breast cancer was included in the national cancer screening program. As emphasized above, breast cancer screening in Korea is ineffective and overdiagnostic. Thus, it is essential to reassess the validity of regular mammographic screening, and the age at which females should commence such screening in Korea.

Our study had several limitations. First, the fact that the work was cross-sectional in nature renders it impossible to draw cause-and-effect conclusions. Second, there may have been a survival bias: if women with a lower SES are more likely to die after being diagnosed with breast cancer, analyses of cancer prevalence would tend to overestimate the correlation between SES and breast cancer. A previous study of Korean breast cancer patients demonstrated that the breast cancer survival rate of those in the highest SES category was 94.1%, while that of those in the lowest SES category was 85.7% [42]. When we reanalyzed our data with consideration of the 8.4% higher cancer prevalence in the lowest versus highest SES quartile, the association between educational level and breast cancer prevalence was robust. Third, breast cancer prevalence was estimated by reference to questionnaire data rather than medical records. Fourth, we lacked data on tumor size or cancer stage.

## Conclusion

The present results demonstrated that the recently increased breast cancer prevalence observed in Korea may be attributable to more effective early detection, rather than to a real increase in breast cancer prevalence, and that breast cancer is, in fact, overdiagnosed. Further prospective studies are needed to explore the correlations between SES, breast cancer incidence, and histopathological data, including tumor size and cancer stage.

## Supporting information

S1 Dataset Survey data. (SAS7BDAT)
